# Clinical Characteristics, Response to Platinum-Based Chemotherapy and Poly (Adenosine Phosphate-Ribose) Polymerase Inhibitors in Advanced Lung Cancer Patients Harboring BRCA Mutations

**DOI:** 10.3390/cancers15194733

**Published:** 2023-09-26

**Authors:** Johnathan Arnon, Michael Tabi, Yakir Rottenberg, Aviad Zick, Philip Blumenfeld, Tamar Hamburger, Eli Pikarsky, Eti Avraham, Leeby Levine, Aron Popovtzer, Tamar Yablonski-Peretz, Luna Kadouri, Hovav Nechushtan

**Affiliations:** 1 Sharett Institute of Oncology, Hadassah Medical Center, Jerusalem 91120, Israelluna@hadassah.org.il (L.K.); hovavnech@hadassah.org.il (H.N.); 2 Factuality of Medicine, Hebrew University of Jerusalem, Jerusalem 12272, Israel; 3 Department of Pathology, Hadassah Medical Center, Jerusalem 91120, Israel; 4 Stern College for Women, Yeshiva University, New York, NY 10033, USA

**Keywords:** nonsmall cell lung cancer (NSCLC), BRCA, platinum chemotherapy, poly (adenosine-phosphate-ribose) polymerase inhibitors (PARPi)

## Abstract

**Simple Summary:**

Carriers of germline BRCA1/2 pathogenic variants are at increased risk of developing BRCA-associated malignancies (breast, ovarian, prostate and pancreatic cancer), which are responsive to treatment with poly (adenosine phosphate-ribose) polymerase inhibitors (PARPi) and platinum-based chemotherapy (PBC). However, the role of BRCA mutations in tumor development and in the prediction of response to treatment in nonsmall cell lung cancer (NSCLC) remains unclear. In this study, we retrospectively analyzed a cohort of 26 patients with NSCLC harboring BRCA mutations treated at our center, demonstrating unique clinical features, including many nonsmokers and BRCA mutations. Importantly, patients with NSCLC and BRCA mutations demonstrate increased sensitivity to PBC and PARPi in comparison to patients with NSCLC and wild-type BRCA. The results suggest that BRCA mutations play some oncogenic role in NSCLC and warrant further prospective trials of treatment of NSCLC harboring BRCA mutations with PARPi.

**Abstract:**

The oncogenic role and clinical relevance of *BRCA* mutations in NSCLC remain unclear. We aim to evaluate the characteristics and clinical outcomes of patients with NSCLC harboring *BRCA* mutations treated at Hadassah Medical Center (HMC). We retrospectively assessed all patients with advanced NSCLC who underwent next-generation sequencing (NGS) and were found to have pathogenic somatic *BRCA* mutations (p-BRCA). We compared clinical outcomes in NSCLC patients with wild-type BRCA (wt-BRCA) matched by age, stage, gender, smoking, PDL-1 and driver mutations. Between 2015 and 2022, we evaluated 598 patients with advanced NSCLC using NGS and found 26 patients with p-BRCA, of whom 17 (65.4%) were carriers of germline BRCA variants and represented 1% of all BRCA carriers HMC. The median age of diagnosis was 67 years old (40–78), 13 patients (50%) had a history of smoking and 9 patients (34.6%) had additional driver mutations (EGFR, ALK, BRAF, MET or ERBB2). Objective response rate and median progression-free survival (PFS) for first-line platinum-based chemotherapy in the p-BRCA group compared to wt-BRCA controls were 72.2% and 16 months (CI 95%, 5–22), compared to 47.4% and 7 months (CI 95%, 5–9), respectively, and HR for PFS was 0.41 (CI 95%, 0.17–0.97). Six patients in the p-BRCA group were treated with advanced-line poly (adenosine-phosphate-ribose) polymerase inhibitors (PARPi), with a durable response observed in four patients (66%). In this cohort, patients with NSCLC harboring p-BRCA exhibit high-sensitivity PARPi and a prolonged response to platinum, suggesting some oncogenic role for BRCA mutations in NSCLC. The results support further prospective trials of the treatment of NSCLC harboring p-BRCA with PARPi.

## 1. Introduction

BRCA1/2 tumor suppressor genes are part of DNA double-strand break repair by homologous recombinant repair [1]. Carriers of pathogenic variations (PV) in BRCA1/2 are at an increased risk of developing BRCA-associated malignancies, including breast, ovarian, pancreas and prostate cancer [2,3]. Tumors harboring BRCA1/2 pathogenic mutations are highly responsive to treatment with poly (adenosine phosphate-ribose) polymerase inhibitors (PARPi) [4,5,6] and platinum-based chemotherapy (PBC) [7].

Locally advanced and metastatic nonsmall cell lung cancer (NSCLC) is an aggressive disease with a 5-year overall survival rate (OS) of less than 10% [8]. Somatic pathogenic or likely pathogenic mutations in BRCA1/2 (p-BRCA) are found in approximately 2–3% of NSCLC cases, the majority of which are not associated with carriers of BRCA PVs. [9,10,11]. Furthermore, only approximately 1% of BRCA carriers develop NSCLC, resembling the prevalence in the general population [3,12,13]. In addition, the majority of patients with NSCLC harboring p-BRCA have a history of smoking, a limited history of other malignancies and present metastatic or advanced disease with adenocarcinoma histology [9]. While PBC remains the backbone of first-line treatment for NSCLC, there are no predictive markers for response to PBC in NSCLC. Additionally, PARPi demonstrated limited efficacy in the treatment of NSCLC [14,15,16,17]. Hence, the oncogenic role of p-BRCA in NSCLC in tumor development and in the prediction of response to treatment remains unclear and testing for BRCA1/2 mutations is not considered to be an integral part of molecular workup in cases of newly diagnosed NSCLC.

We previously reported a case series including 10 patients with advanced NSCLC and germline or somatic mutations in BRCA1/2 with increased sensitivity to PBC and prolonged overall survival (OS) [18]. We aim to further evaluate the characteristics and clinical outcomes of NSCLC patients harboring p-BRCA treated at our center. Here, we report an expanded cohort of 26 patients with NSCLC and p-BRCA and describe in more detail their genetic characteristics and clinical outcomes. Importantly, we show that these patients exhibit high sensitivity to PARPi and a prolonged response to PBC, suggesting that, at least in some of these cases, BRCA mutations have an oncogenic role.

## 2. Materials and Methods

### 2.1. Participants and Data Collection

We retrospectively evaluated our pathology data bases for all patients with locally advanced (stage IIIB or IIIC) or metastatic NSCLC who were treated at Hadassah Medical Center (HMC) between January 2015 and December 2022, underwent tumor-tissue or liquid-based ct-DNA next-generation sequencing (Foundation-one©, Oncomine©, Tempus© or Gaurdiant©) and were found to have p-BRCA. We included in our analysis only patients with BRCA1/2 mutations classified as pathogenic or likely pathogenic according to ClinVar or Varsom [19,20], who received treatment (according to national compressive network guidelines) for at least 3 months and were followed for at least one year for surviving patients or earlier in case of death. Patients were determined to be carriers of BRCA1/2 if they had confirmed independently tested germline PV or ct-DNA-based analyses with a variant allele frequency (VAF%) of at least 40%. Data regarding demographic characteristics, personal and family medical history, pathological and genetic results, as well as treatment and medical outcomes, was retrospectively retrieved from patient records.

We then used our pathology data bases to build a control group of NSCLC patients with wildtype BRCA1/2 (wt-BRCA) treated at HMC matched by age of diagnosis (5-year difference allowed), stage at diagnosis, gender, smoking, programmed death ligand 1 (PDL-1) status group (<1%, 1–49% or >50%) and the following actionable mutations: epidermal growth factor (EGFR), anaplastic lymphoma kinase translocations (ALK), B-Raf (BRAF), Erb-B2 Receptor Tyrosine Kinase 2 (ERBB2) and mesenchymal-epithelial transition factor (MET).

### 2.2. Study Endpoints

The primary endpoints were progression-free survival (PFS) of treatment with PBC or PARPi in the p-BRCA group independently and as compared to the wt-BRCA control group, defined as the interval between start of treatment and progression of disease or death, and best response under these treatments according to RESICT 1.1 criteria. The secondary endpoint was OS of the p-BRCA group independently and, as compared to the wt-BRCA control group, defined as the interval between the date of diagnosis and death due to any cause. Surviving patients were censored at the date of the last follow-up.

### 2.3. Statistical Analysis

Descriptive analyses were carried out using the median and confidence interval (CI 95%) or range for quantitative variables and percentages for qualitative variables. Baseline characteristics were compared between p-BRCA group and matched wt-BRCA control group using independent sample *t*-test for continuous variables and chi-square test for categorical variables. OS and PFS medians and rates were analyzed using the Kaplan–Meier method and compared between the cohort and matched control groups by the log-rank test. Hazard ratios (HR) were estimated using Cox’s proportional hazards regression with control group as the reference group. All tests were two-sided. The statistical analysis was conducted using IBM SPSS© version 25.

## 3. Results

### 3.1. Patient Characteristics

Between 2015 and 2022, we evaluated 598 patients with locally advanced or metastatic NSCLC using comprehensive tumor-tissue (513 patients) or liquid-based ct-DNA (85 patients) next-generation sequencing (NGS). Thirty-six patients were found to have p-BRCA, constituting 6.0% of all NSCLC patients tested. Of them, 26 patients were treated for at least 3 months, had sufficient data for follow-up for a minimum of one year for surviving patients or earlier in case of death and were included in the final analysis. Seventeen patients (65.4%) were confirmed carriers of corresponding germline BRCA1/2 PVs, of which 14 patients (53.8%) were carriers of classical Ashkenazi-Jewish PVs (BRCA2 617delT, BRCA1 185delAG or BRCA1 5382incC), representing approximately 1% (17 of 1641) of all registered BRCA1/2 carriers at HMC.

The median age of diagnosis was 67 years old (range 40–78); only 13 patients (50%) had a history of smoking, and 9 patients (34.6%) had a history of other malignancies, mainly breast cancer. Pathology for 24 patients (92.3%) revealed adenocarcinoma and 9 patients (34.6%) had additional mutations in actionable driver mutations (EGFR, ALK, BRAF, MET or ERBB2). Two patients (7.7%) who were confirmed carriers of BRCA had additional somatic pathogenic mutations in BRCA1/2.

From the 598 patients with NSCLC that were evaluated using NGS panels, we located an additional 26 patients with wt-BRCA matched to the p-BRCA group by age of diagnosis, stage at diagnosis, gender, smoking status, PDL-1 group and major driver mutations (Table 1). The median age of diagnosis for the wt-BRCA group was 68.5 years old (range 40–79). Thirteen patients had a history of smoking, and only 3 patients (88.5) in the wt-BRCA had a history of other malignancies. Pathology for 25 patients (96.2%) in the wt-BRCA group revealed adenocarcinoma and 9 patients (34.6%) had additional mutations in actionable driver mutations (EGFR, ALK, BRAF, MET or ERBB2) (Table 1).

### 3.2. Treatment and Clinical Outcomes

In both p-BRCA and wt-BRCA groups, the age, stage, gender, smoking, PDL-1 status, driver mutations, and tumor histology of first-line treatments were balanced. Patients with locally advanced disease (stage IIIB) were all treated with concurrent PBC with definitive radiation. Six patients (23.1%) in each group were treated with first-line targeted therapies for mutations in EGFR, ALK or BRAF. First-line immunotherapy was added to PBC in the treatment of 10 patients (42.3%) in the p-BRCA group and 14 patients (53.8%) in the wt-BRCA group (Table 1).

**Figure 1:**
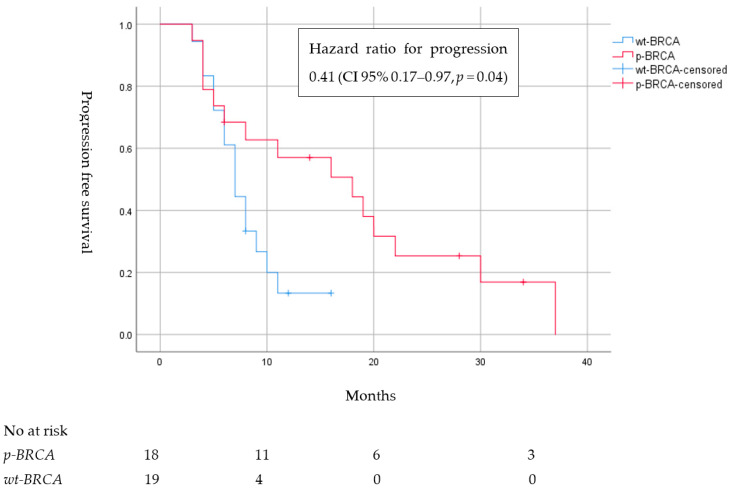
Progression-free survival of platinum-based chemotherapy in patients with locally advanced or metastatic nonsmall cell lung cancer harboring pathogenic mutations in *BRCA1/2* (p-BRCA) compared to control group with wildtype BRCA (wt-BRCA) matched by gender, satge, age, smoking status, programmed death ligand group and additional driver mutations.

Eighteen patients (69.3%) in the p-BRCA group, all of whom were without mutations in EGFR, ALK or BRAF, were treated with first-line PBC, with an objective response rate (ORR) of 72.2% and a median PFS of 16 months (CI 95% 5–22), compared to an ORR of 47.4% and median PFS of 7 months (CI 95%, 5–9) in the wt-BRCA control group, HR for platinum PFS 0.41 (CI 95% 0.17–0.97, *p* = 0.04). (Table 2 and Figure 1).

**Figure 2:**
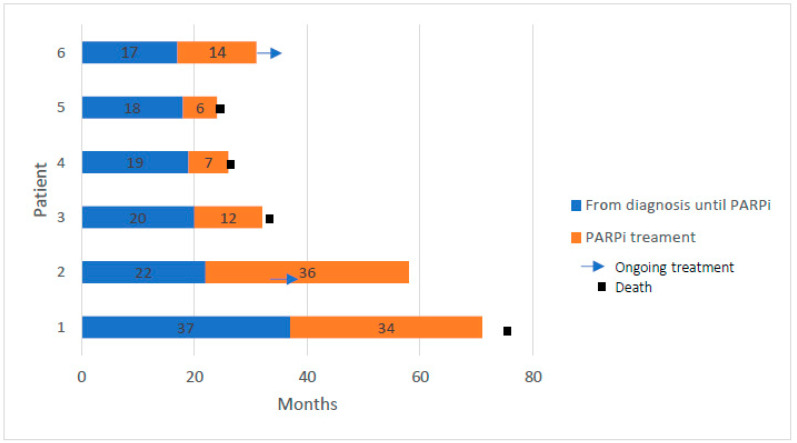
Duration of response to initial treatment and subsequent poly (adenosine phosphate-ribose) polymerase inhibitors (PARPi) treatment in 6 patients with nonsmall cell lung cancer harboring pathogenic *BRCA1/2* mutations.

Six patients (23.1%) in the p-BRCA group were treated with second- or third-line PARPi, with an objective response observed in four patients (66.6%), a median PFS of 13 months (range 6–36) and two patients achieving a durable response for 3 years (Figure 2). These six patients, at a median age of 65.5 years old (range 54–75), all had adenocarcinoma histology, were without additional driver mutations and all had suffered disease progression after PBC, including three patients with central nervous system progression before PARPi treatment.

**Figure 3:**
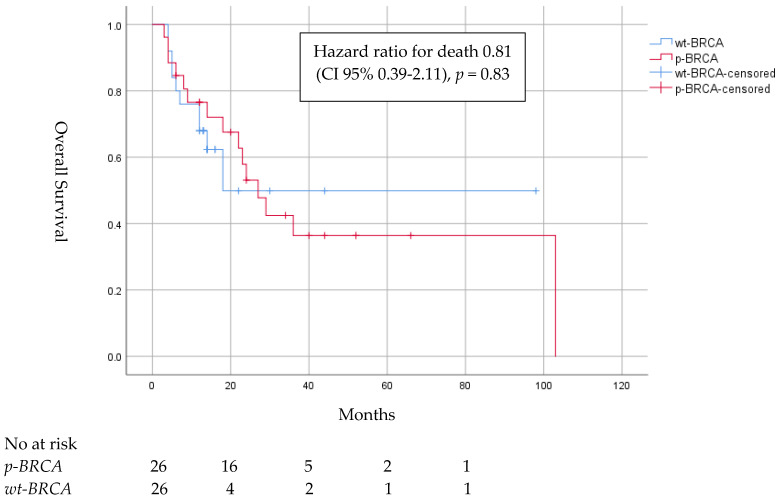
Overall survival of patients with locally advanced or metastatic nonsmall cell lung cancer harboring pathogenic mutations in *BRCA1/2* (p-BRCA) compared to control group with wildtype BRCA (wt-BRCA) matched by gender, age, satge, smoking status, programmed death ligand group and additional actionable mutations.

Median OS for the p-BRCA group compared to wt-BRCA was 22.5 months (CI 95% 12–34) and 13.0 months (CI 95% 12–14), respectively, and HR for death was 0.81 (CI 95% 0.39–2.11). Three years after diagnosis, eight patients (30.8%) in the p-BRCA survived compared to three patients (11.5%) in the wt-BRCA group (Figure 3).

## 4. Discussion

In this study, we presented the clinical and genetic characteristics of 26 patients with NSCLC harboring mutations in BRCA1/2 and showing increased sensitivity to PBC and PARPi. The prevalence of NSCLC patients harboring p-BRCA out of all NSCLC in our database was approximately three times higher than reported in large-scale molecular studies and according to the cBioPortal database [9,11,21]. This is likely due to the over-representation of the Ashkenazi-Jewish population at our center, where the estimated carrier frequency is 2.5% [22]. Indeed, 53.8% of patients in our study were carriers of classical Ashkenazi-Jewish PVs and the prevalence of p-BRCA in NSCLC patients in our study is similar to a recently published cohort of 445 patients with NSCLC treated in a different center in Israel [23]. The fact that only 1% of BRCA carriers at HMC developed NSCLC, together with the relatively older age of diagnosis (median 67 years old), corresponds with previous findings suggesting that BRCA carriers are not at increased risk for developing NSCLC [3,12,13]. However, it is important to note that a relatively large fraction of patients (13 of 26) in our cohort were nonsmokers, most of whom were BRCA carriers, which is greatly different from the findings of previous studies in which the majority of NSCLC cases harboring p-BRCA were smokers and non-BRCA carriers [9,10,11]. Thus, while the relative risk of developing lung cancer in the general population is mainly influenced by smoking, our findings suggest that the BRCA mutation may have some role in the development of NSCLC, perhaps more significant for nonsmokers.

Notably, nine patients (34.6%) in our study had oncogenic driver mutations. Mutations in oncogenic drivers are found in approximately 15–20% of patients with NSCLC, more so in nonsmokers with adenocarcinoma histology presenting with advanced disease. Comutations in tumor suppressor genes such as TP53, RB1, LKB1 or BRCA1/2 have been described as manifestations of early clonal selection in up to 50% of cases of NSCLC harboring driver mutations, especially in EGFR-mutated tumors [24]. Hence, the relatively high proportion of patients with oncogenic driver mutations in our study can be attributed to the fact that four patients (15.4%) had oncogenic driver mutations, had de-novo somatic pathogenic BRCA comutations and were not carriers of BRCA, together with an over-representation of nonsmoking patients with adenocarcinoma histology and advanced disease at presentation. Comutations in tumor suppressor genes affect tumor proliferation and the microenvironment and are responsible for acquired resistance to targeted treatments, leading to a relatively poor prognosis in cases of NSCLC harboring driver mutations and comutations in TSG [24]. Nevertheless, the six patients in the p-BRCA group in our cohort who had driver mutations in EGFR, ALK or BRAF and were treated with first-line targeted therapies all responded to these therapies with a PFS of 23 months (range 12–30), questioning whether somatic BRCA mutations influence response to targeted therapies.

To analyze the response and survival of patients with NSCLC and p-BRCA to PBC, we carefully selected a group of patients with wt-BRCA and similar clinical and pathologic characteristics. Patients with p-BRCA showed significantly longer PFS and response to PBC compared to wt-BRCA controls [median PFS of 16 months (CI 95% 5–22), compared to 7 months (CI 95%, 5–9), respectively, HR 0.41 (CI 95% 0.17–0.97, *p* = 0.04), and ORR of 72.2% compared to 47.4%, respectively (*p* = 0.12)] and compared to results from first-line platinum-immunotherapy trails such as Keynote-189 (ORR 47.6% median PFS 8.8 months) [25] and 9LA (ORR 38% median PFS 6.7 months) [26]. Of note, PDL-1 status and first-line concurrent immunotherapy treatments were well balanced between the groups to lower the confounding effect of immunotherapy, as this treatment has a significant effect on both ORR and PFS when added to first-line PBC in correlation to PDL-1 status [25,26].

Importantly, six patients in the p-BRCA group were treated with advanced-line PARPi, with a tumor response observed in four cases and a long-term response of approximately 3 years in two cases. Of note, these six patients all exhibited prolonged PFS and responses to first-line PBC, corresponding with the recently published PIN study, in which NSCLC patients who responded to first-line PBC benefited from PARPi maintenance therapy [27]. The difference in median OS between the p-BRCA and wt-BRCA groups was noticeable but not significant, probably due to the small sample size of the heterogeneous cohort consisting of a large proportion of patients who were treated with first-line targeted therapies for ALK, EGFR or BRAF and since half of the patients were still alive at the time of data analysis.

An important issue is whether, in cases of NSCLC, aberrations in BRCA play a role in tumor development and act as oncogenic drivers. In BRCA-associated malignancies, tumor development is dependent on biallelic inactivation, additional somatic mutations or loss of heterozygosity (LOH), which can be measured by homologue recombinant deficiency signatures (HRD) [10,28]. These changes predict tumor sensitivity to PBC and PARPi by means of synthetic lethality [7,28,29]. This HRD phenotype is not considered to be typical of non-BRCA-associated malignancies, suggesting that, in these tumors, BRCA has a neutral oncogenic effect [29,30]. Nevertheless, recent analyses of two large pan-cancer databases (Foundation© and TCGA©) have shown LOH and an HRD-phenotype in at least 20–25% of NSCLC harboring BRCA mutation cases [10,30] and in two cases in which platinum-based chemotherapy caused BRCA reversion mutations [31], suggesting at least a partial oncogenic role for BRCA aberrations in NSCLC. In addition, epigenetic silencing of wildtype BRCA allele may have functional implications both regarding the emergence of the tumor and its response to therapy, as recently demonstrated by a specific micro-mRNA profile in the blood of BRCA carriers, implying that even a deficiency in one BRCA allele may be sufficient to induce tumor development [32].

The fact that, in our cohort, p-BRCA patients exhibited increased sensitivity to PBC and PARPi supports the assumption that, in at least some cases of NSCLC, mutations in BRCA play an oncogenic role and predict response to these treatments. These results also concur with the recent publication by Tschernichovsky et al., which showed prolonged PFS in first-line therapy for seven patients with NSCLC harboring BRCA mutations compared to an institutional cohort [23]. Two BRCA carriers in our cohort had additional somatic BRCA mutations; further HRD testing of tumor tissue samples, especially in cases in which sensitivity to PBC and PARPi was demonstrated, might shed light on the role of BRCA mutations in NSCLC. In addition, the much more widespread availability of HRD testing in NGS panels suggests that such testing could help in the selection of NSCLC patients with p-BRCA for treatments with PARPi, and further prospective trials of treatment of NSCLC harboring p-BRCA with PARPi seem warranted.

This study has several limitations. The small sample size and retrospective design warrant caution when interpreting the findings. While the p-BRCA and wt-BRCA were well balanced in terms of demographics, predictive factors and treatment types, other important factors, such as performance status, were lacking and might have influenced the results. Finally, the molecular analysis relied on multiple commercial panels and methods (ct-DNA and tumor tissue). On the other hand, variant pathogenicity was carefully determined using validated databases and carrier status was determined using independent germline testing or high ct-DNA VAF%.

## 5. Conclusions

In this cohort, patients with NSCLC harboring p-BRCA show high sensitivity to PARPi and a prolonged response to PBC. In addition, these patients have unique clinical features, including a high proportion of nonsmokers with additional driver mutations. While limited to a small sample, the findings suggest that mutations in BRCA play an oncogenic role in NSCLC and predict responses to PBC and, interestingly, to PARPi. The results support further prospective trials testing for BRCA1/2 mutations in NGS panels of NSCLC patients and treatment with PBC and PARPi for patients with p-BRCA and in cases of carriers of BRCA who develop NSCLC. Further molecular analysis, in particular HRD-signature, is required to determine the exact oncogenic role of p-BRCA in NSCLC.

## Figures and Tables

**Table 1:** Clinical characteristics of locally advanced or metastatic nonsmall cell lung cancer patients with somatic pathogenic mutations in BRCA1/2 (p-BRCA) and matched control group (according to age, stage, gender, smoking status, programmed death ligand group (PDL-1) and additional driver mutations) with wildtype BRCA (wt-BRCA) treated at Hadassah Medical Center between January 2015 and December 2022.

Characteristic	p-BRCA Group (*n* = 26)	wt-BRCA Control (*n* = 26)	*p*-Value
Age at Diagnosis—Median (range)	67.0 (40–78)	68.5 (40–79)	0.61
Gender—No. (%) Female Male	11 (42.3) 15 (57.7)	11 (42.3) 15 (57.7)	1.0
Ethnic Origin—No. (%) Ashkenazi-Jewish Non-Ashkenazi-Jewish Arab-Muslim Other	16 (61.5) 8 (30.8) 2 (7.7) 0 (0)	10 (38.5) 8 (30.8) 7 (26.9) 1 (3.8)	0.25
Smoking status—No. (%) Yes—current or former smoker Never smoker	13 (50) 13 (50)	13 (50) 13 (50)	1.0
Personal history of malignancy—No. (%) • Yes - Breast Cancer - Prostate Cancer - Other Cancers • No	• 9 (34.6) - 4 (15.4) - 2 (7.7) - 3 (11.5) • 17 (65.4)	• 3 (11.5) - 0 - 0 - 3 (11.5) • 23 (88.5)	0.048
Stage at diagnosis—No. (%) Locally advanced (IIIB) Metastatic (IV)	4 (15.4) 22 (84.6)	5 (19.2) 21 (80.8)	0.71
Tumor histology—No. (%) Adenocarcinoma Squamous cell carcinoma	24 (92.3) 2 (7.7)	25 (96.2) 1 (3.8)	0.65
BRCA Mutation—No. (%) BRCA 2 6174delT 7007G>T Others BRCA 1 185delAG 5382insC Others	20 (76.9) 10 (38.5) 2 (7.7) 8 (30.8) 6 (23.1) 3 (11.5) 1 (3.8) 2 (7.7)	0 0	n/a
Confirmed BRCA germline carrier—No. (%) Yes No Not available	17 (65.4) 5 (19.2) 4 (15.4)	0	n/a
Somatic driver mutations—No. (%) EGFR Exon 19 deletion or Exon 21 L858R BRAF V600E ERBB2 mutation MET Exon 14 skipping mutation ALK-EML translocation	4 (15.4) 1 (3.8) 2 (7.7) 1 (3.8) 1 (3.8)	4 (15.4) 1 (3.8) 2 (7.7) 1 (3.8) 1 (3.8)	1.0
PDL-1 status group—No. (%) >50% 1–49% <1% Not reported	7 (26.9) 6 (23.1) 11 (42.3) 2 (7.7)	7 (26.9) 6 (23.1) 13 (50) 0 (0)	0.94
First-line Treatment Platinum-based chemoradiation With subsequent immunotherapy Without subsequent immunotherapy Platinum-based chemotherapy With concurrent immunotherapy Without concurrent immunotherapy Targeted therapy Immunotherapy single agent Other chemotherapy	4 (15.4) 2 (7.8) 2 (7.8) 14 (53.8) 9 (34.6) 5 (19.2) 6 (23.1) 1 (3.8) 1 (3.8)	5 (19.2) 4 (11.5) 1 (3.8) 14 (53.8) 10 (38.5) 4 (11.5) 6 (23.1) 1 (3.8) 0 (0)	0.96

**Table 2:** Clinical characteristics, best tumor response and median progression-free survival in locally advanced or metastatic nonsmall cell lung cancer patients with somatic pathogenic mutations in BRCA1/2 (p-BRCA) and matched control group with wildtype BRCA (wt-BRCA) treated with first-line platinum-based chemotherapy (PBC) at Hadassah Medical Center between January 2015 and December 2022. Of note, all patients treated with first-line PBC were without mutations in ALK, EGFR or BRAF.

Treatment Type	p-BRCA Group (*n* = 18)	wt-BRCA Control (*n* = 19)	*p*-Value
Age at Diagnosis—Median (range)	67.0 (40–75)	67.0 (40–78)	0.98
Gender—No. (%) Female Male	7 (38.9) 11 (61.1)	9 (47.4) 10 (52.6)	0.40
Smoking status—No. (%) Yes—current or former smoker Never smoker	12 (66.6) 6 (33.3)	10 (52.6) 9 (47.4)	0.38
Stage at diagnosis—No. (%) Locally advanced (IIIB) Metastatic (IV)	4 (22.2) 14 (77.8)	5 (26.3) 14 (73.7)	0.77
Tumor histology—No. (%) Adenocarcinoma Squamous cell carcinoma	17 (94.4) 1 (5.6)	19 (100) 0	0.97
PDL-1 status group—No. (%) >50% 1–49% <1%	4 (22.2) 6 (33.3) 8 (44.4)	6 (31.6) 5 (26.3) 8 (42.1)	0.79
First-line Treatment—No. (%) PBC with radiation With subsequent immunotherapy Without subsequent immunotherapy PBC without radiation With concurrent immunotherapy Without concurrent immunotherapy	4 (22.2) 2 (11.1) 2 (11.1) 14 (77.8) 9 (50.0) 5 (27.8)	5 (26.3) 4 (21.0) 1 (5.3) 14 (73.7) 10 (52.6) 4 (21.1)	0.77
Best tumor response to first-line PBC—No. (%) • Objective response - Complete response - Partial response • Stable disease • Progressive disease	• 13 (72.2) - 5 (27.8) - 8 (44.4) • 2 (11.1) • 3 (16.7)	• 9 (47.4) - 3 (15.8) - 6 (31.6) • 5 (26.3) • 5 (26.3)	0.12
Median progression-free survival to first-line PBC—months (CI 95%)	16 (5–22)	7 (5–9)	0.04

## Data Availability

The data presented in this study are available upon request from the corresponding author. The data are not publicly available due to privacy.
